# Synthesis of Graphene Based Membranes: Effect of Substrate Surface Properties on Monolayer Graphene Transfer

**DOI:** 10.3390/ma10010086

**Published:** 2017-01-21

**Authors:** Feras Kafiah, Zafarullah Khan, Ahmed Ibrahim, Muataz Atieh, Tahar Laoui

**Affiliations:** 1 Department of Mechanical Engineering, King Fahd University of Petroleum & Minerals, Dhahran 31261, Saudi Arabia; fkafiah399@gmail.com (F.K.); aiibrahim@kfupm.edu.sa (A.I.); 2 Department of Mechanical Design and Production Engineering, Zagazig University, Zagazig 44519, Egypt; 3 Qatar Environment and Energy Research Institute, HBKU, Qatar Foundation, P.O. Box 5825, Doha, Qatar; mhussien@qf.org.qa

**Keywords:** graphene transfer, graphene membrane, microfiltration membrane, hydrophobic, surface roughness

## Abstract

In this work, we report the transfer of graphene onto eight commercial microfiltration substrates having different pore sizes and surface characteristics. Monolayer graphene grown on copper by the chemical vapor deposition (CVD) process was transferred by the pressing method over the target substrates, followed by wet etching of copper to obtain monolayer graphene/polymer membranes. Scanning electron microscopy (SEM), atomic force microscopy (AFM), and contact angle (CA) measurements were carried out to explore the graphene layer transferability. Three factors, namely, the substrate roughness, its pore size, and its surface wetting (degree of hydrophobicity) are found to affect the conformality and coverage of the transferred graphene monolayer on the substrate surface. A good quality graphene transfer is achieved on the substrate with the following characteristics; being hydrophobic (CA > 90°), having small pore size, and low surface roughness, with a CA to RMS (root mean square) ratio higher than 2.7°/nm.

## 1. Introduction

Graphene as a carbon-based nanomaterial is attractive from the standpoint of science and technology due to its exceptional properties. It is very strong (100 times stronger than steel) [1,2], highly conductive (charge carrier mobility ~200,000 cm^2^/Vs, which is higher than that of copper) [3], has a high surface area (2630 m^2^/g) [4], is highly thermally conductive (~5000 W/mK, which is 10 times greater than copper) [5,6], is highly transparent (absorbs only 2.3% of incident light) [7,8], and flexible [9,10,11].

With the aforementioned unusual properties, graphene opens doors for many applications across disciplines. It is used in electronic applications as transistors [12], chemical and biosensors [13], transparent conducting electrodes [14,15], optoelectronics [16,17,18], and in medical applications such as tissue engineering [19] and drug delivery [20]. It is found in energy applications in both generation fields such as solar cells [21], fuel cells [22], and storage fields such as supercapacitors [23], hydrogen storage [24], and rechargeable batteries [25]. Environmental applications mainly involve water purification [26].

Various fabrication routes exist for graphene production, including the mechanical [27,28] and chemical exfoliation [29,30,31] of high-quality graphene; direct growth on metal or carbide substrates using the chemical vapor deposition process (CVD) [32,33,34]; and chemical routes via graphene oxide and unzipping of carbon nanotubes [35].

At present, no single fabrication route that produces graphene sheets is suitable for all potential applications, as every route has its advantages and disadvantages [36,37]. Graphene grown onto copper (Cu) by chemical vapor deposition has been found to be the most commonly used among the other preparation processes [38]. The basic principle of the CVD process is to decompose a carbon-based gas using heat to provide a source of carbon that can then re-arrange to form graphene over a catalyst substrate [39]. The CVD process is cost effective, and not only yields reasonably high quality but also offers a large area of graphene [36,40,41,42] and the sheets produced can be transferred to other substrates or used directly in an application [43,44,45,46].

Currently, graphene transfers onto desired substrates using various methods are implemented in two ways: the wet transfer method and the dry transfer method. The most straightforward wet transfer method is to etch the metal away chemically to obtain free floating graphene composites that can be scooped onto chosen substrates. Other well-known wet etching methods include the standard transfer method [43], the direct transfer method [47], and roll-to-roll transfer [48].

In all wet transfer methods, the Cu substrate is removed by a wet etching process using a copper etchant such as ferric chloride [32], iron nitrate [45], or ammonium persulfate (APS) [48]. To minimize the cracking/tearing of transferred films, it is important to ensure good adhesion between the target substrate and the transferred graphene layer. The roughness of the substrate and its hydrophobicity control the adhesion of the graphene film.

Bunch et al. [49], reviewed recent theoretical advances in the understanding of how graphene adheres and conforms to different substrates. Three parameters were found to affect the quality of transferred graphene, namely surface roughness, porosity, and wettability (degree of hydrophobicity). The substrate surface should be smooth to have good contact between the graphene and substrate, and the pore size should be as small as possible to provide a good support for graphene which may otherwise tear and crack during the transfer process. The surface should be hydrophobic to keep the graphene/substrate interface non-wetted by the etchant solution which may otherwise damage and detach the graphene layer from the substrate [50].

In the present work, we examine the effect of substrate surface characteristics in terms of pore size, wettability (degree of hydrophobicity), and surface roughness on the graphene transferability experimentally. We selected eight polymeric substrates with different surface characteristics and transferred monolayer CVD grown graphene on copper foils onto these polymeric substrates. Copper was removed by wet etching to obtain monolayer graphene/polymer composite membranes. Two conditions related to the substrate surface were found to affect the quality of transferred graphene. Firstly, the substrate should have an adequate hydrophobicity (contact angle (CA) > 90°); if the first condition is achieved, then the ratio of the surface contact angle to the root mean square (RMS) value should be roughly higher than 2.5. Otherwise, poor graphene quality will be obtained. The quality of graphene was checked by Field Emission Scanning Electron Microscope (FESEM) and Atomic Force Microscopy (AFM) characterization, and an ionic transport study of potassium chloride (KCl) through the graphene/substrate composite membranes.

## 2. Materials and Methods

### 2.1. Materials

Table 1 lists the commercial substrates used in this study. We purchased the polypropylene (PP) Ultrafiltration membrane from Sterlitech Co., Kent, WA, USA, and three polyvinylidene difluoride (PVDF) nanofiltration membranes with three different pore sizes (10, 20, and 100 nm) and polyethersulfone (PES) nanofiltration membranes from Novamem Advance Separations Company, Switzerland. All these membranes were used as substrate supports for monolayer graphene transfer. Although PVDF substrates (1), (2), (3), and (4) that are listed in Table 1 have the same pore size (10 nm), according to the manufacturer, they have different pore structures and surface wettability. The CVD monolayer graphene was procured from ACS Material Company, Medford, MA, USA. Raman spectroscopy was performed and confirmed the coverage of monolayer graphene over the Cu substrate, see Figure S1. The APS etchant (used to dissolve Cu during the graphene transfer process) was prepared by mixing 5% (w/v) of ammonium persulfate (APS) (Eurostar Scientific Ltd., Liverpool, UK) with de-ionized water. Potassium chloride (KCl), used for diffusion studies, was purchased from Merck group chemicals, Germany.

### 2.2. Monolayer Graphene Transfer Process

**Figure 1:**
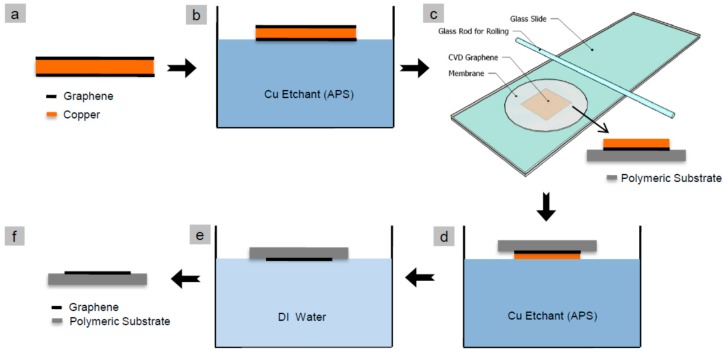
Monolayer graphene transfer process to polymeric substrates: (**a**) commercial monolayer chemical vapor deposition (CVD) graphene with graphene on both sides of the Cu foil; (**b**) removal of graphene from one side of the foil by floating over ammonium persulfate (APS) copper etchant for 10 min; (**c**) Cu/graphene attachment to the polymeric substrate by sandwiching between two glass slides and gentle press rolling with the glass rod; (**d**) Copper removal by wet etching process using APS etchant for 2 h; (**e**) Graphene/substrate washing process by two de-ionized baths for 10 min each; (**f**) air dried graphene/substrate composite membrane.

We transferred monolayer graphene onto various polymeric substrates by modifying the direct transfer method developed by Regan et al. [47]. It is a simple method capable of transferring large graphene areas with the lowest possible defects. The as received CVD monolayer graphene was initially floated over the APS copper etchant for 10 min to remove graphene from one side (Figure 1b) which usually grows on both sides of the copper foil during the CVD process. The Cu/graphene/polymeric substrate was then sandwiched between two glass slides and gently pressed using a glass rod (Figure 1c). The new sandwiched composite was then floated over copper etchant for almost 2 h to remove the copper layer leaving behind the graphene monolayer attached onto the polymeric substrate (Figure 1d). The graphene/substrate was then washed with two de-ionized water baths for 10 min each to remove all the traces of residual etchant that may have remained within the graphene/polymer substrate assembly. The assembly (graphene/polymer composite membrane) was finally air dried.

### 2.3. Ionic Transport Study

**Figure 2:**
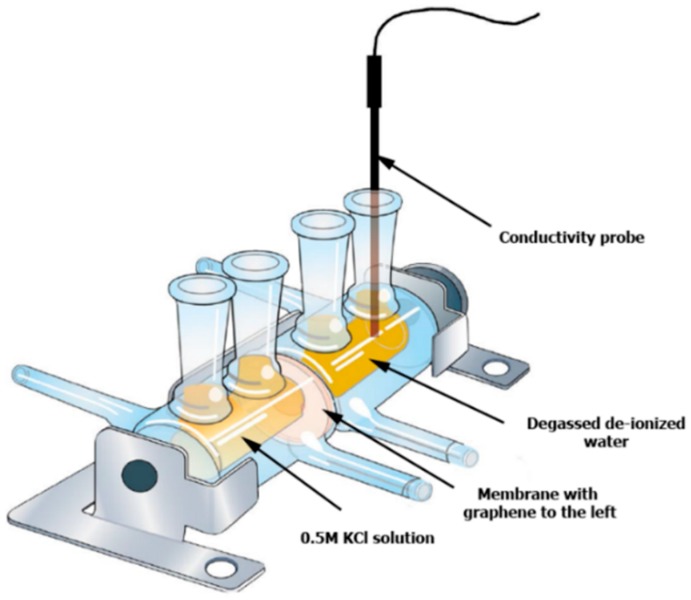
Side-Bi-Side diffusion cell used to study ionic transport.

The quality of transferred graphene was checked by studying the ionic transport through the graphene/substrate composite. To do so, a special Side-bi-Side glass diffusion cell procured from Permegear Inc., Hellertown, PA, USA was used (Figure 2). The cell is composed of two glass chambers having the same volume capacity (7 mL for each chamber). The two chambers are clamped together and sealed tightly together across a 3 mm interfacing orifice where the membrane is placed to conduct the ionic transport studies. This method was adapted from the work of Sean et al. [51].

Both cell chambers are throughly cleaned by de-ionized water and air dried before the composite graphene membrane is placed between them. The active side of the composite membrane (graphene side) faces the left chamber. The cell is then tightened using a rubber screw. Both sides of the membrane are then washed with ethanol to remove any water bubbles close to the membrane surfaces. To remove any entrapped ethanol from the previous stage, the left cell side was washed with 0.5 M KCl solution and the right side was washed three times with de-gassed de-ionized water.

The ionic transport study was performed with 7 mL of 0.5 M KCl solution. KCl solution was introduced into the left chamber of the cell and 7 mL de-ionized water was introduced into the right chamber. Both solutions were magnetically stirred during the diffusion process to minimize concentration polarization effects. Potassium and chloride ions diffused through the graphene membrane towards the de-ionized water side. The diffusion rate of the ions was measured by monitoring the change in conductivity over time, using an eDAQ conductivity isoPod electrode (eDAQ Pty Ltd., Denistone East, New South Wales, Australia) dipped in the de-ionized water side of the diffusion cell, as shown in Figure 2. Conductivity was recorded every 15 s for 10 min. The slope of the conductivity-time curve was measured and compared with the same slope for the as received bare substrate (without graphene layer). The percentage of the ion blockage was calculated by dividing the difference between the slopes of the conductivity-time curves for the bare and composite membranes over the slope of the bare membrane.

## 3. Results and Discussion

FESEM, AFM, and water wettability characterizations for the as received polymer membranes and the graphene/polymer membranes were carried out to understand the role of the substrate surface characteristics on the graphene transferability as well as its coverage and quality.

**Figure 3:**
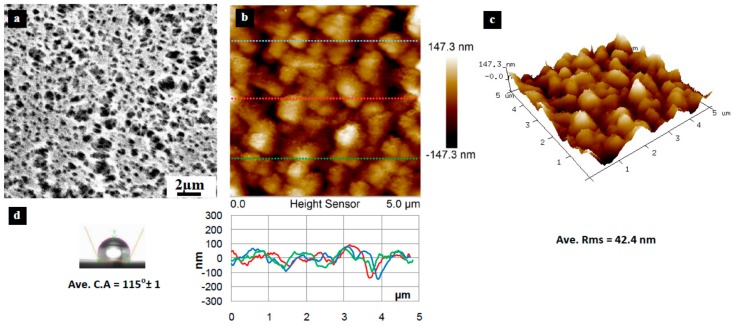
Surface characteristics of the as received PP substrate (100 nm pore size), (**a**) SEM micrograph; (**b**) 5 × 5 µm^2^ AFM image (top) and three section profiles (bottom) with an average RMS equal to 42.4 nm; (**c**) 3D profile for the selected area; (**d**) surface contact angle (CA) with an average equal to 115° ± 1°.

**Figure 4:**
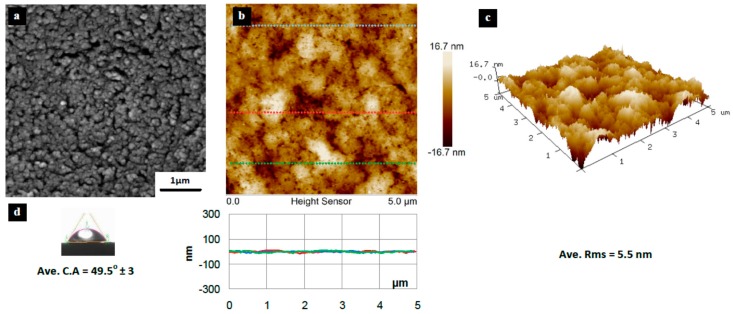
Surface characteristics of as received PES substrate (20 nm pore size), (**a**) SEM micrograph; (**b**) 5 × 5 µm^2^ AFM image (top) and three section profiles (bottom) with an average RMS equal to 5.5 nm; (**c**) 3D profile for the selected area; (**d**) surface contact angle (CA) with an average equal to 49.5° ± 3°.

**Figure 5:**
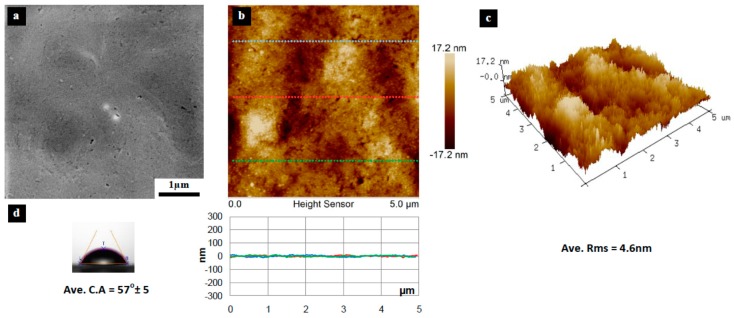
Surface characteristics of as received PVDF 1 substrate (10 nm pore size), (**a**) SEM micrograph; (**b**) 5 × 5 µm^2^ AFM image (top) and three section profiles (bottom) with an average RMS equal to 4.6 nm; (**c**) 3D profile for the selected area; (**d**) surface contact angle (CA) with an average equal to 57° ± 5°.

**Figure 6:**
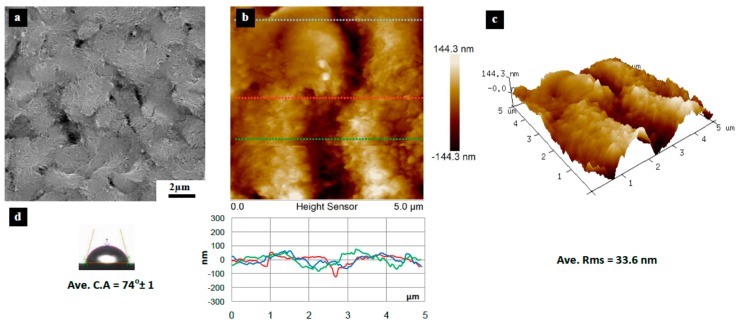
Surface characteristics of as received PVDF 2 substrate (10 nm pore size), (**a**) SEM micrograph; (**b**) 5 × 5 µm^2^ AFM image (top) and three section profiles (bottom) with an average RMS equal to 33.6 nm; (**c**) 3D profile for the selected area; (**d**) surface contact angle (CA) with an average equal to 74° ± 1°.

**Figure 7:**
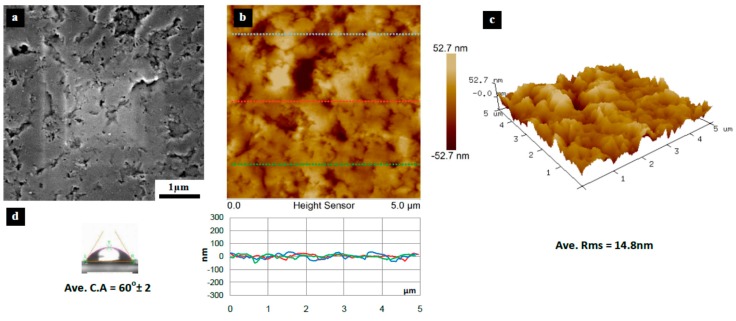
Surface characteristics of as received PVDF 3 substrate (10 nm pore size), (**a**) SEM micrograph; (**b**) 5 × 5 µm^2^ AFM image (top) and three section profiles (bottom) with an average RMS equal to 14.8 nm; (**c**) 3D profile for the selected area; (**d**) surface contact angle (CA) with an average equal to 60° ± 2°.

**Figure 8:**
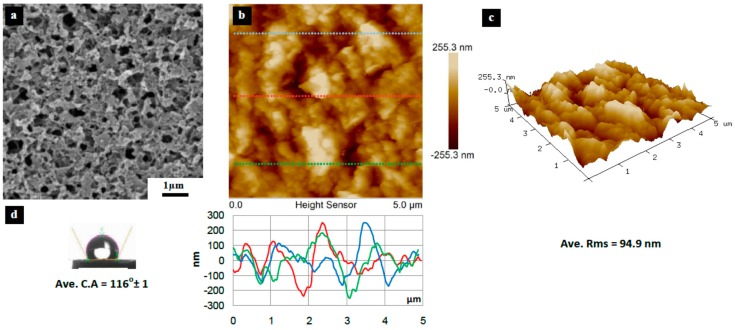
Surface characteristics of as received PVDF 4 substrate (10 nm pore size), (**a**) SEM micrograph; (**b**) 5 × 5 µm^2^ AFM image (top) and three section profiles (bottom) with an average RMS equal to 94.9 nm; (**c**) 3D profile for the selected area; (**d**) surface contact angle (CA) with an average equal to 116° ± 1°.

**Figure 9:**
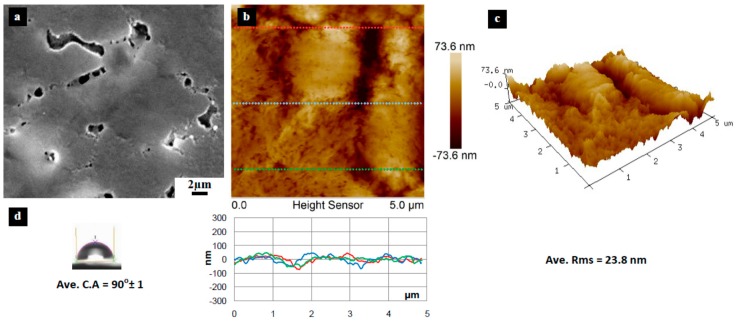
Surface characteristics of as received PVDF 5 substrate (20 nm pore size), (**a**) SEM micrograph; (**b**) 5 × 5 µm^2^ AFM image (top) and three section profiles (bottom) with an average RMS equal to 23.8 nm; (**c**) 3D profile for the selected area; (**d**) surface contact angle (CA) with an average equal to 90° ± 1°.

**Figure 10:**
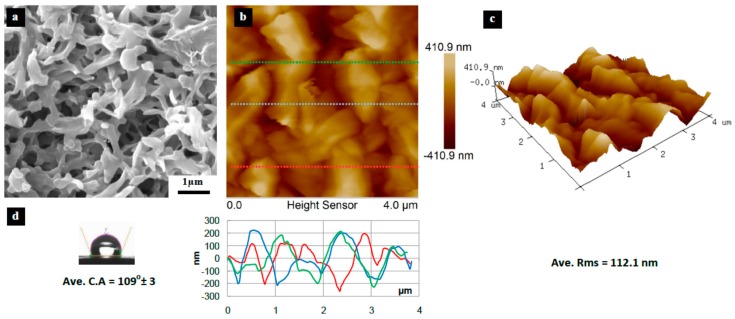
Surface characteristics of as received PVDF 6 substrate (100 nm pore size), (**a**) SEM micrograph; (**b**) 5 × 5 µm^2^ AFM image (top) and three section profiles (bottom) with an average RMS equal to 112.1 nm; (**c**) 3D profile for the selected area; (**d**) surface contact angle (CA) with an average equal to 109° ± 3°.

**Figure 11:**
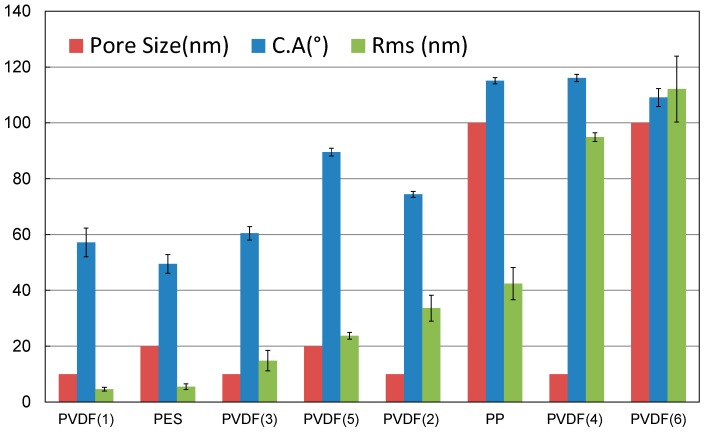
Substrate surface characteristics: pore size, contact angle (CA), and surface roughness (RMS value).

Figure 3, Figure 4, Figure 5, Figure 6, Figure 7, Figure 8, Figure 9, Figure 10 and Figure 11 show FESEM micrographs, AFM images, and contact angle measurement results for all bare substrates (before graphene transfer). FESEM was used to explore the surface morphology which was then followed by AFM characterization to check surface roughness within a selected area of 5 × 5 µm^2^. However, for PVDF 6 substrate, an area of 4 × 4 µm^2^ was used as AFM tip instability problems were encountered due to a rather rough surface for this particular substrate. Three sections were taken to explore the surface profile and to calculate the average root mean square (RMS) values. 3D surface profiles were also captured to check and qualitatively validate surface roughness values.

The AFM results show that the PVDF 6 substrate with 100 nm pore size was found to have the roughest surface of RMS = 112 nm and appreciably high surface wettability of CA = 109° as shown in Figure 10. This should be expected since the PVDF 6 substrate has a large pore size and a network structure as revealed by the SEM image. Among the commercial PVDF membranes, the PVDF 1 substrate with a pore size of 10 nm exhibits the smoothest surface (RMS = 4.6 nm) and a very low contact angle (CA = 57°), as clearly evidenced by the SEM image shown in Figure 5.

Figure 11 summarizes the surface characteristics for all the substrates considered in this study. The contact angle increases slightly with an increase in the surface roughness. This is consistent with other findings [52,53], which report that water wettability is related to the surface roughness. The surface becomes increasingly more hydrophobic with increasing surface roughness.

It is not always necessarily true that smaller pore size substrates should have smoother surfaces. PVDF 4 shown in Figure 8 has a 10 nm pore size (according to the manufacturer) and yet shows a higher roughness (RMS = 94.9 nm) when compared to the other 10 nm pore size PVDF substrates (PVDF 1, 2, and 3). This has to be related with the substrate cross-sectional porosity and pore structure, i.e., it has a porous surface (active side) and a smoother surface (other/opposite side).

Based on the contact angle measurements, four out of the eight substrates, namely, PES, PVDF 1, PVDF 2, and PVDF 3 are considered hydrophilic; (CA < 90°), and the remaining are considered hydrophobic.

### Graphene Transfer onto Polymeric Substrates

We transferred monolayer graphene onto polymeric substrates using the procedure (pressing method) shown in Figure 1. We could categorize the transferability into three different categories according to the transfer outcome (as shown in Table 2) as (a) no transfer at all; (b) good quality transfer; and (c) poor quality transfer. No transfer (or failed transfer) is encountered when the copper/graphene detached from the polymeric substrate during the copper etching step, as shown in Figure 1d. The good and poor transfer refer to successful graphene deposition on the polymeric substrate, but with low defects (tears and cracks) or with a high degree of such defects, respectively.

**Figure 12:**
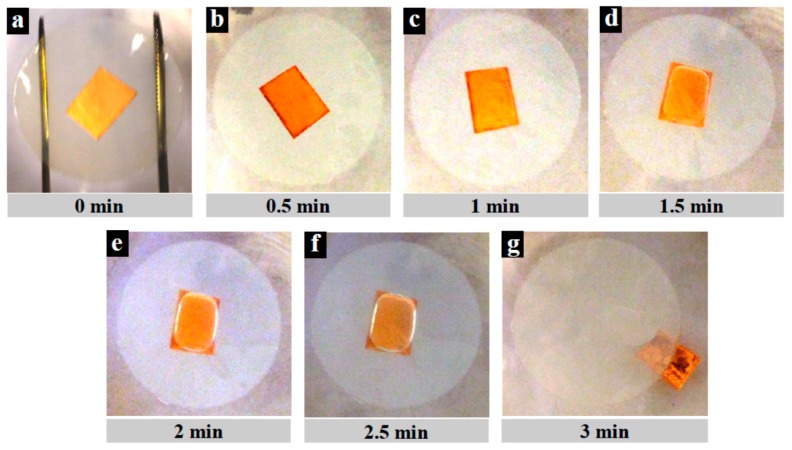
Copper/graphene and PVDF 1 substrate detachment process during copper etching step in transfer process, (**a**) Cu/graphene attached to substrate and floated over APS etchant; (**b**) after 30 s, etchant starts to penetrate between copper/graphene and substrate from the edges (dark regions); (**c**) after 60 s; (**d**) air bubbles become entrapped between Cu/graphene and the substrate; (**e**) after 2 min, air bubble becomes enlarged (**f**) air bubble tries to cover the entire attachment area; (**g**) Cu/graphene and substrate detachment is completed.

Graphene detached from four substrates (PES, PVDF 1, PVDF 2 and PVDF 3) during copper etching as mentioned earlier (failed transfer). The contact angle of these substrates is lower than 90° and as such they can be considered hydrophilic. Figure 12 illustrates the detachment of copper/graphene and the substrate. Their surfaces therefore wet easier as compared to hydrophobic surfaces and this allows the APS etchant to penetrate between the polymeric substrate and the Cu/graphene causing graphene detachment. The etching process starts with floating the substrate/graphene/Cu composite over an etchant as shown in Figure 12a. After 30 s, the etchant liquid starts to wet the polymeric substrate and penetrates between the substrate and graphene/Cu (Figure 12b). This process continues with time as shown in Figure 12c, where after approximately 90 s, a large air bubble forms between the substrate and graphene/Cu (Figure 12d). The air bubble continues to enlarge and finally causes detachment. This process was the same for all four substrates mentioned above. The more hydrophilic the substrate is, the quicker the detachment.

To prevent graphene layer detachment, a polymeric substrate with an adequate degree of hydrophobicity is required to prevent etchant penetration during the etching process that usually takes around 2 h.

The other four substrates (PP, PVDF 4, PVDF 5, and PVDF 6) have the required degree of hydrophobicity that prevents the detachment process; PVDF 2 has an average CA = 90° and PVDF 4 has a CA = 116°. Two of the four substrates (PP and PVDF 5) have smoother surfaces compared to the PVDF 4 and PVDF 6. The PP has a surface roughness with a root mean square (RMS) value = 42.4 nm; PVDF 5 has an RMS value = 23.8 nm. The other two substrates have a rougher surface, with 94.9 nm and 112.1 nm RMS values for PVDF 4 and PVDF 6, respectively (Table 2).

**Figure 13:**
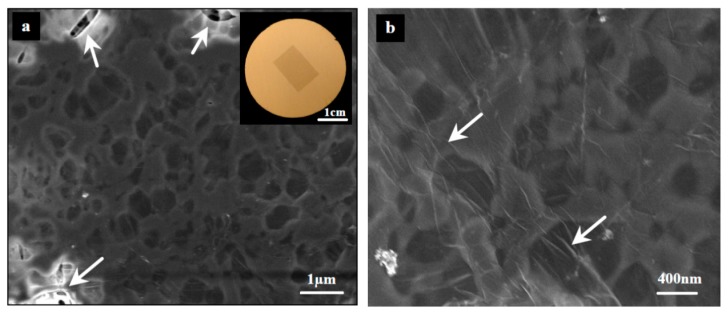
FESEM micrograph of transferred graphene (~1 × 1 cm^2^) onto PP substrate. The white arrow on the left image indicates the tears in the graphene upon transfer [54].

**Figure 14:**
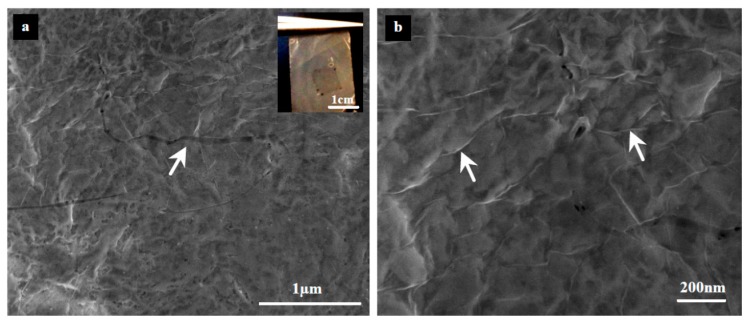
FESEM micrograph of transferred graphene (~1 × 1 cm^2^) onto PVDF 5 substrate. The white arrow on the left image indicates the tears in the graphene upon transfer [54].

Surface roughness has a critical impact on the quality of transferred graphene. Figure 13 and Figure 14 show SEM micrographs for the successfully transferred graphene over PP and PVDF 5 substrates, respectively. An approximately 1 × 1 cm^2^ of monolayer graphene can be clearly seen by the naked eye at the upper right corner (inset). The FESEM images (Figure 13a and Figure 14a) reveal a transferred graphene with good quality but with some defects (tears and cracks) as indicated by the white arrows. Higher magnification FESEM images (Figure 13b and Figure 14b) show a well-known phenomenon for 2D materials, called wrinkles, that are indicated by arrows. Wrinkles are considered as proof of existence of graphene.

**Figure 15:**
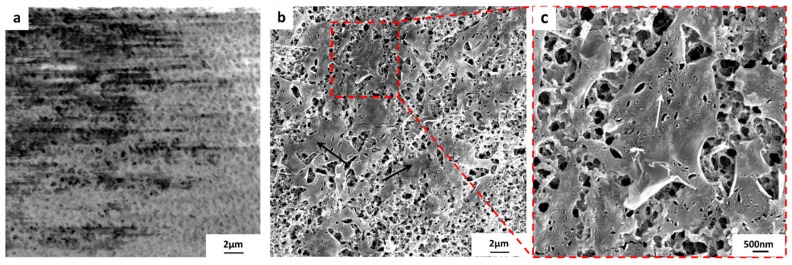
FESEM micrographs of the transferred graphene (~1 × 1 cm^2^) onto PVDF 4 substrate, (**a**) because the graphene is discontinuous (therefore, unable to conduct away electrons), artifacts are present even at low beam current; (**b**) FESEM of coated graphene/PVDF 4 composite, arrows indicate the discontinuous graphene batches; (**c**) high magnification FESEM micrograph shows the tears within the graphene layer and also shows the PVDF 4 substrate pores underneath.

**Figure 16:**
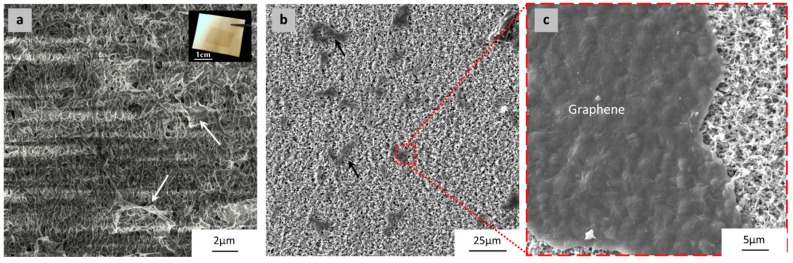
FESEM micrographs of the transferred graphene (~1 × 1 cm^2^) onto PVDF 6 substrate. (**a**) because the graphene is discontinuous (therefore, unable to conduct away electrons), artifacts are present even at low beam current; (**b**) FESEM of coated graphene/PVDF 6 composite, arrows indicate the discontinuous graphene batches; (**c**) high magnification FESEM micrograph shows one of the graphene domains and the PVDF 6 substrate structure underneath.

Figure 15 and Figure 16 show FESEM micrographs of poor quality graphene transferred onto PVDF 4 and PVDF 6, respectively. The graphene tore and cracked due to the high surface roughness of both substrates. It should be pointed out that the FESEM samples were not coated by any conducting film for obtaining images shown in Figure 15a and Figure 16a. The blurring noted in these figures is due to the loss of charging caused by the discontinuation of graphene areas, which is proof of the poor quality of the graphene. The FESEM samples were then coated with 10 nm of platinum ions, and the discontinuous batches of graphene were seen, as shown in Figure 15b,c and Figure 16b,c. PVDF 6 exhibited a severe tearing of graphene compared to PVDF 4. This could be attributed to the high surface roughness of both substrates.

A high surface roughness causes poor adherence between the graphene and the substrate surface; this will facilitate the detachment and forms a discontinuity between graphene domains as shown in Figure 15 and Figure 16.

Transport of KCl ions through the four graphene/substrate membranes was performed to check and confirm the quality of transferred graphene. A simple diffusion cell was used for this purpose in which a 0.5 M KCl solution is contained in the cell's left side chamber and de-ionized water in the right side chamber. Due to the concentration difference (caused by osmosis), the KCl ions are transported from the left side toward the right side through the graphene/polymer composite membrane. The rate of ion transport is calculated by monitoring the change of de-ionized water conductivity over time. Based on the fact that a defect free graphene is impermeable even to very small ionic species such as helium [55,56], the quality of the transferred graphene can be determined from the extent of ion leakage (or ion blockage) through the membrane.

**Figure 17:**
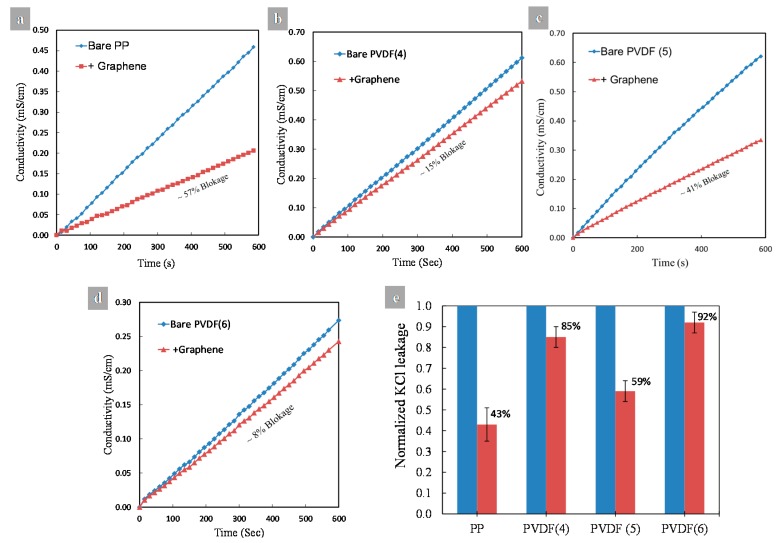
Ionic transport measurements of KCl ions passing through substrates before and after graphene transfer, change of conductivity with time for (**a**) PP substrate; (**b**) PVDF 4 substrate; (**c**) PVDF 5 substrate; (**d**) PVDF 6 substrate; (**e**) normalized KCl ions leakage for all substrates.

Figure 17 shows the KCl ionic transport measurements in terms of conductivity with time for all polymer substrates before and after graphene transfer. The percentage of ion blockage can be easily calculated using the slopes of the curves for each substrate. PP and PVDF 5 blocked 57% and 41% of KCl ions, respectively (Figure 17a,c). On the other hand, PVDF 4 and PVDF 6 blocked only 15% and 8% of KCl ions, respectively (Figure 17b,d). These results suggest that the quality of the transferred graphene on PP and PVDF 5 is better than that of PVDF 4 and PVDF 6. They also provide evidence of the presence of transfer-process-induced defects in the graphene layer.

So far, two conditions related to substrate surface characteristics should be controlled in order to have a good quality (low defect density) graphene transfer. First, the substrate surface should have a reasonable hydrophobicity (contact angle > 90°) and second, the contact angle (CA) to surface roughness (RMS value) should be higher than 2.7 (CA/RMS > 2.7°/nm).

A hydrophobic surface along with a high CA/RMS ratio means the substrate has the required hydrophobicity to prevent penetration of the etchant between the Cu/graphene and substrate, and the low roughness helps provide better contact between the transferred graphene and substrate, and minimize the unsupported graphene domains that could be detached during the etching process [50].

## 4. Conclusions

Graphene transfer to a working substrate is the key to a wide range of graphene applications. This paper explores the effect of substrate surface characteristics, mainly the surface roughness and wettability (degree of hydrophobicity) on graphene transferability. To transfer graphene with high quality, the substrate surface should be smooth to allow the graphene to conform and adhere with minimum tears and cracks, and should have high hydrophobicity to prevent etchant penetration between the graphene and substrate during copper etching, which would otherwise cause graphene detachment. CVD monolayer graphene was transferred to eight different polymeric substrates having different surface characteristics by a simple pressing method, followed by wet etching of copper using an APS etchant. Graphene failed to transfer over four substrates (PES, PVDF 1, PVDF 2, and PVDF 3) because they were hydrophilic, which facilitated etchant penetration between the Cu/graphene and the substrate and caused graphene detachment. Graphene successfully transferred to the other four substrates that were hydrophobic (PP, PVDF 4, PVDF 5, and PVDF 6). PP and PVDF 5 exhibited better graphene transferability in terms of quality, due to the lower surface roughness as compared to PVDF 4 and PVDF 6. The quality of the graphene was checked by FESEM characterization and the simple diffusion of potassium chloride ions (KCl) through the transferred graphene/substrate membrane. To obtain high-quality graphene, the substrate should have an adequate hydrophobicity (contact angle (CA) > 90°); if this is attained, then the ratio of the contact angle value to the root mean square (RMS) value should be higher than 2.7 (CA/RMS > 2.7°/nm).

## Figures and Tables

**Table 1:** Polymeric substrate characteristics. (PP: polypropylene; PES: polyethersulfone; PVDF: polyvinylidene difluoride).

No.	Substrate	Pore Size (nm)	Thickness (µm)	Surface Wetting	pH Range
1	PP	100	75–110	Hydrophobic	1–14
2	PES	20	20	Hydrophobic	2–12
3	PVDF 1	10	50	Hydrophobic	0–12
4	PVDF 2	10	50	Hydrophobic	0–12
5	PVDF 3	10	50	Hydrophobic	0–12
6	PVDF 4	10	50	Hydrophobic	0–12
7	PVDF 5	20	25	Hydrophobic	0–12
8	PVDF 6	100	125	Hydrophobic	N/A

**Table 2:** Summary of Graphene transferability onto different polymeric substrates.

No.	Substrate	Pore Size (nm)	CA (°)	RMS (nm)	CA/RMS	Graphene Transfer	Graphene Quality	Reason
1	PP	100	115 ± 1.1	42.4 ± 5.8	2.7	Yes	Good	Low roughness
2	PES	20	50 ± 3.3	5.5 ± 1.0	9.2	Failed	N/A	N/A
3	PVDF 1	10	57 ± 5.1	4.6 ± 0.7	12.4	Failed	N/A	N/A
4	PVDF 2	10	74 ± 1.1	33.6 ± 4.7	2.2	Failed	N/A	N/A
5	PVDF 3	10	60 ± 2.4	14.8 ± 3.7	4.1	Failed	N/A	N/A
6	PVDF 4	10	116 ± 1.3	94.9 ± 1.6	1.2	Yes	Poor	High roughness
7	PVDF 5	20	90 ± 1.4	23.8 ± 1.2	3.8	Yes	Good	Low roughness
8	PVDF 6	100	109 ± 3.2	112.1 ± 11.8	1.0	Yes	Poor	High roughness

## Supplementary Materials

The following are available online at www.mdpi.com/1996-1944/10/1/86/s1. Figure S1: Raman spectroscopy for the as received CVD monolayer graphene.
