# Supplementary material for: Synthesis of Graphene Based Membranes: Effect of Substrate Surface Properties on Monolayer Graphene Transfer
Source: Materials (Basel). 2017 Jan 21;10(1):86. doi: 10.3390/ma10010086 (PMC5344567; doi:10.3390/ma10010086)
Supplement: Supplementary file 1 [file materials-10-00086-s001.pdf]

Supplementary Materials: Synthesis of Graphene Based Membranes: Effect of Substrate Surface Properties on Monolayer Graphene Transfer

Feras Kafiah, Zafarullah Khan, Ahmed Ibrahim, Muataz Atieh and Tahar Laoui

Raman Spectroscopy of the Commercial Monolayer Graphene

Monolayer graphene was purchased from ACS Material Company, USA. Raman spectroscopy was performed to confirm whether the graphene was monolayer or multilayer. The pure copper sample was also tested to see if there was any noise background in the spectrum coming from the copper, as shown in Figure S1.

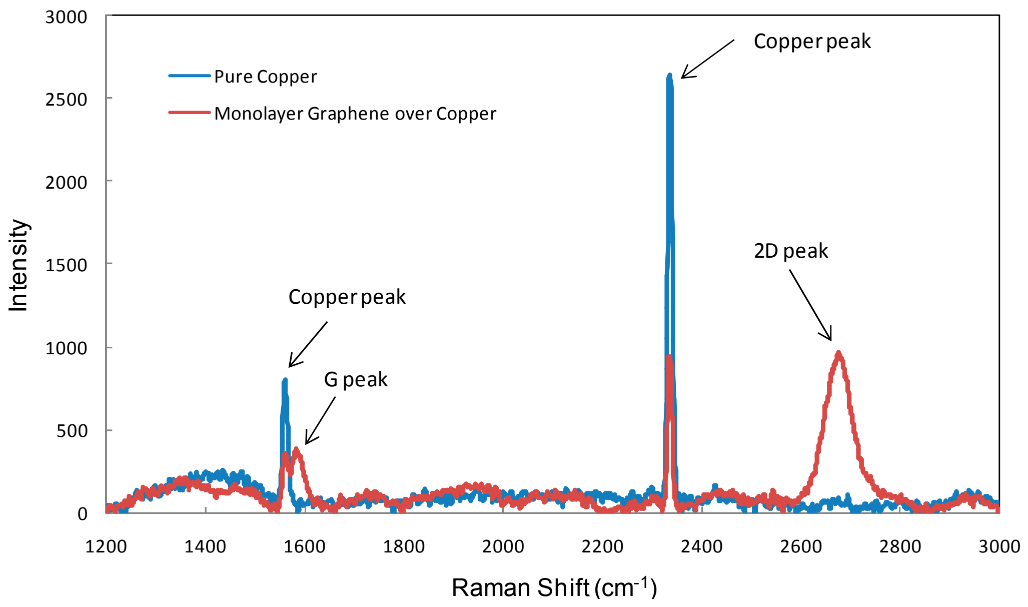

Figure S1. Raman spectroscopy for the as received CVD monolayer graphene.

The two most important features in the Raman fingerprint of graphene are the G peak that is at ~1580 cm^{-1} and the 2D peak at ~2670 cm^{-1} [1,2]. The other peaks shown in Figure S1 belong to the copper that carries the graphene; this can be seen clearly on the copper Raman spectrum. Monolayer graphene shows 2D peak intensity that is roughly 2–4 times higher than the G peak. The intensity of the G peak increases almost linearly as the graphene thickness increases [3]. Since the 2D peak is almost three times the G peak as shown in Figure S1, the CVD graphene purchased is monolayer.

References

1. Ferrari, A.C.; Meyer, J.C.; Scardaci, V.; Casiraghi, C.; Lazzeri, M.; Mauri, F.; Piscanec, S.; Jiang, D.; Novoselov, K.S.; Roth, S.; et al. The Raman fingerprint of graphene. *arXiv* **2006**, arXiv:cond-mat/0606284.
2. Allen, M.J.; Tung, V.C.; Kaner, R.B. Honeycomb carbon: A review of graphene. *Chem. Rev.* **2009**, *110*, 132–145.
3. Ni, Z.; Wang, Y.; Yu, T.; Shen, Z. Raman spectroscopy and imaging of graphene. *Nano Res.* **2008**, *1*, 273–291.
